# Comparative Analysis of Machine Learning Techniques for Heart Rate Prediction Employing Wearable Sensor Data

**DOI:** 10.3390/sports13030087

**Published:** 2025-03-13

**Authors:** Asieh Namazi, Ehsan Modiri, Suzana Blesić, Olivera M. Knežević, Dragan M. Mirkov

**Affiliations:** 1 Department of Physical Education and Sport Science, Iran University of Science and Technology (IUST), Tehran 16846-13114, Iran; anamazi@iust.ac.ir; 2 Helmholtz Centre for Environmental Research–UFZ, Department of Computational Hydrosystems, Permoserstrasse 15, 04318 Leipzig, Germany; 3 Institute for Medical Research, University of Belgrade, 11000 Belgrade, Serbia; 4 Faculty of Sport and Physical Education, University of Belgrade, 11000 Belgrade, Serbia; olivera.knezevic@fsfv.bg.ac.rs (O.M.K.); dragan.mirkov@fsfv.bg.ac.rs (D.M.M.)

**Keywords:** heart rate prediction, wearable sensors, machine learning, singular spectrum analysis, health monitoring

## Abstract

Monitoring heart rate (HR) is vital for health management and athletic performance, and wearable technology enables scientists to obtain real-time cardiovascular insights. This study compares Machine Learning (ML) techniques, including Long Short-Term Memory (LSTM) networks, Physics-Informed Neural Networks (PINNs), and 1D Convolutional Neural Networks (1D CNNs). Then, we develop a hybrid Singular Spectrum Analysis (SSA)-Augmented ML technique to predict HR using wearable sensor data. Additionally, we investigate the impact of incorporating auxiliary physiological inputs, such as breathing rate (BR) and RR intervals, on predictive accuracy. The study utilizes the cardiorespiratory data acquired through wearable sensors while practising sports, including 126 recordings from 81 participants (53 males, 28 females) engaged in 10 different sports. Physiological signals were collected at 1 Hz using the BioHarness 3.0 (Zephyr Technology, Mangaluru, India). The dataset includes individuals with varied levels of sports experience (beginner, intermediate, and advanced), allowing for a more comprehensive evaluation of HR variability across different expertise levels. Our results demonstrate that the hybrid SSA-LSTM model reaches the lowest prediction error by effectively capturing HR dynamics. Furthermore, integrating HR, BR, and RR data significantly enhances accuracy over single or dual parameter inputs. These findings support adopting multivariate machine learning models for health monitoring, improving HR prediction accuracy for fitness and preventive healthcare.

## 1. Introduction

Heart rate (HR) monitoring has recently become a key component in health and fitness management, particularly in sports and athletic performance [1,2]. The dawn of wearable technology has transformed the collection and analysis of physiological data, providing remarkable real-time insights into an individual’s cardiovascular state [3,4,5]. This technological leap has enhanced our understanding of human physiology during physical effort. Additionally, it has opened new opportunities for proactive health management and risk prevention, especially for athletes and fitness enthusiasts [6]. The importance of accurate HR monitoring and prediction in sports cannot be overstated. For athletes, understanding and anticipating changes in HR can differentiate between optimal performance and potentially dangerous overexertion [2]. Athletes, trainers, coaches and scientists can fine-tune training regimens, optimize match performance, and prevent cardiovascular emergencies by accurately anticipating HR trends that could arise from extreme physical stress [7,8]. Traditional HR prediction approaches, such as linear autoregressive (AR) and autoregressive-moving average (ARMA) models, have provided foundational procedures but often fall short due to the nonlinear and nonstationary nature of physiological time series data [9,10,11]. Consequently, researchers have been increasingly exploring more advanced prediction techniques that can better capture the complexity of HR dynamics. Methods following AutoRegressive Integrated Moving Average (ARIMA), Random Forest (RF), Support Vector Machines (SVM), Kalman Filters, Singular Spectrum Analysis (SSA), Copula-based analysis, and various neural networks have been applied to HR prediction with varying degrees of success [12]. Despite the progress made, significant room for improvement remains, particularly regarding accuracy, adaptability, and computational efficiency for real-time applications in wearable gadgets. Wearable devices, including chest straps, smartwatches, and specialized sports monitoring kits, have become ubiquitous among professionals and amateurs [13]. These instruments capture physiological data with inconsistent accuracy depending on the instrument type and measurement method. Chest strap monitors, which use electrocardiogram (ECG) technology, are generally considered more precise for HR measurements, especially during intense activities or movement [14]. ECG data from chest straps provide accurate heart rate (HR) information and insights into R-R intervals, which represent the time between consecutive heartbeats and serve as a key measure of the sinus heart period in chronological or heartbeat order [15]. Variations in RR reflect both the vagal and sympathetic modulation of the heart’s sinus node. They are commonly used for heart rate variability analysis, a non-invasive tool for assessing autonomic function. While some advanced chest strap devices can estimate breathing rate (BR) through specialized algorithms, this capability is not universal and may require additional validation [16]. Smartwatches, which typically employ optical sensors (Photoplethysmography), can provide convenient continuous monitoring but may be less accurate during particular activities [17]. When collected accurately, combining these data attributes can contribute to a comprehensive understanding of an individual’s physical state during training, potentially leading to better reliable HR prediction models [3]. Recent machine learning (ML) advancements, primarily deep learning, have enabled evolved models capable of bearing physiological time series data sophistication [18]. For instance, Long Short-Term Memory (LSTM) networks have demonstrated strong capabilities in capturing long-term dependencies in time series, making them particularly well-suited for HR prediction [19,20]. Additionally, innovations such as Physics-Informed Neural Networks (PINNs), which incorporate physical constraints into the network to adhere to known physiological laws, and one-dimensional Convolutional Neural Networks (1D CNNs), adapted for time series analysis, have shown notable promise in HR prediction tasks [21,22,23]. Furthermore, hybrid models combining CNNs with LSTMs have successfully enhanced predictive accuracy by capturing both spatial and temporal features in ECG signals [24]. Bidirectional LSTM (BiLSTM) networks also enhance model accuracy by seizing both forward and backward dependencies in time series. When integrated with attention mechanisms, they can focus on essential temporal features, improving performance and reducing complexity [25,26]. Additional advanced methods, such as Evolutionary Neural Networks, have been explored to optimize predictions dynamically, especially for fitness and sports applications [27]. The following challenges still persist in HR prediction despite the mentioned advancements:

1. HR data variability is high, influenced by individual differences and outer elements, complicating precise prediction [28,29].
2. Integrating HR with other indicators, such as BR and RR intervals, improves accuracy but increases model complexity [30].
3. Achieving accurate predictions in varying environmental conditions and for heterogeneous populations remains challenging, as physical activity and fitness level variability affect HR prediction accuracy [31].
4. Balancing prediction accuracy with the processing power constraints of wearable devices is crucial for real-time applications, especially in low-resource settings [32].
5. Ensuring models perform well across different physical activities and populations without specific recalibration is crucial for broader applicability [31].

This study proposes hybrid models that combine SSA with different machine-learning approaches to address these challenges. SSA decomposes time series data into trend, periodic, and noise components, which may enhance the model’s ability to handle the nonlinear and nonstationary characteristics of HR data [12,33,34]. Our study aims to grasp the temporal dynamics of HR with improved robustness by associating SSA with ML methods. Additionally, considering various physiological factors (HR, BR, and RR intervals) could offer a better understanding of an individual’s cardiovascular status.

The specific objectives of this study are as follows:

1. Evaluate and compare the performance of ML models in predicting HR using ECG-derived data.
2. Assess the impact of incorporating additional physiological parameters (BR and RR intervals) on the accuracy of HR predictions [28].
3. Investigate the effectiveness of a hybrid approach in improving HR prediction accuracy and robustness.
4. Explore the potential of these predictive models for real-time health monitoring and risk prevention in sports and fitness contexts [29,30].
5. Analyze the computational efficiency and practicality of implementing these models in wearable devices for on-the-go predictions [32].

By addressing these goals, this investigation contributes to a growing body of knowledge at the intersection of wearable technology, sports science, and AI. These findings will have significant consequences for athletes, coaches, healthcare providers, and device manufacturers, providing them with critical insights to optimize performance and improve personalized healthcare solutions [5]. Integrating the advanced capabilities of AI and ML with the abundance of data generated by wearable sensors, we strive to develop health monitoring systems that are faster, more accurate, more reliable, and more useful than their current counterparts, paving the way towards novel approaches for physical activity and cardiovascular health in the next few years [3].

## 2. Data and Methodology

### 2.1. Data Collection and Preprocessing

We used the “Sport Database: Cardiorespiratory data acquired through wearable sensors while practicing sports” dataset, developed by [35], which provides a comprehensive collection of cardiorespiratory data recorded during various physical activities. This dataset includes recordings from 81 subjects practising ten different sports, each recording capturing HR, BR, and RR intervals. The data were gathered using the BioHarness 3.0 by Zephyr (www.zephyranywhere.com, accessed on 10 March 2025), a wearable chest strap device known for accurately measuring physiological signals, including ECG signals at a sampling rate of 250 Hz and automatically computing HR and RR intervals and BR signals at a sampling rate of 1 Hz, across diverse physical activities. Since our study aimed to predict these physiological signals at the same frequency, no additional resampling or frequency adjustments were applied. This study follows a structured experimental design aimed at evaluating machine learning models for HR prediction using physiological time-series data. The approach consists of the following steps:

- Collect HR, BR, and RR interval data using a validated wearable sensor across multiple sports disciplines.
- Apply statistical methods to remove outliers, normalize physiological signals, and extract relevant features.
- Train and test machine learning models, including SSA-augmented LSTM, CNN, PINN, and RNN architectures.
- Compare models based on MAE, assessing the impact of SSA and auxiliary inputs (BR and RR) on HR prediction accuracy.
- Discuss the implications for real-time health monitoring and future model improvements.

**Figure 1:**
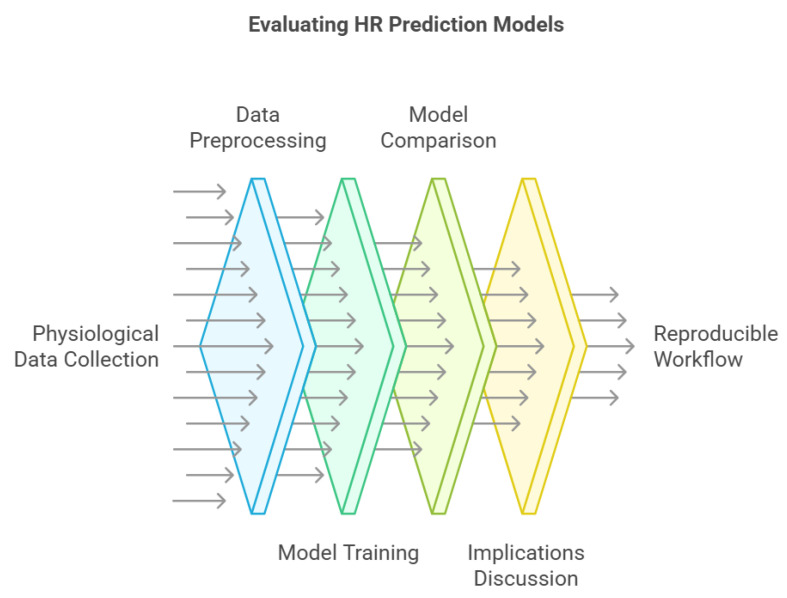
Overview of the HR prediction model evaluation process, highlighting key stages from data collection to reproducible workflow.

This workflow ensures reproducibility, allowing researchers to replicate or extend the study by adjusting the input features in the future, by applying alternative ML models or validating the findings using different datasets. Figure 1 provides an overview of the HR prediction model evaluation process, illustrating the key steps from data acquisition to model assessment and reproducibility.

Participant demographic details, including age, gender, height, weight, smoking habits, alcohol consumption, and weekly training frequency, are summarized in Table 1. The surveys are provided contextual information alongside the physiological recordings. These data support analyzing how demographic factors impact cardiorespiratory responses to exercise.

We applied preprocessing strategies to enrich data reliability, employing statistical thresholds to drop outliers in the HR, BR, and RR time series. We also normalized these physiological time series to compare them consistently across subjects and sports. Additionally, our preprocessing addressed discrepancies in sampling frequency that arose from the diversity of sports and participant behaviours. This dataset offers a solid basis for creating HR prediction models across various exercise intensities. It is a valuable resource for studying cardiorespiratory adaptations in various sports and advancing algorithms that allow the real-time health monitoring and prediction of sudden cardiac events in athletic settings [35].

### 2.2. A Hybrid Models to Predict HR

In this study, we developed a hybrid approach by integrating ML, deep learning (DL), and SSA techniques to improve HR prediction accuracy.

#### Singular Spectrum Analysis (SSA)

SSA is a non-parametric technique used for time series analysis, particularly for modelling the deterministic components of complex data. It effectively supports trend detection, de-noising, forecasting, and change-point detection. In HR prediction, SSA models the deterministic elements of the time series, separating underlying trends and periodic components from random oscillations, which provides a solid foundation for HR prediction.

The SSA method includes the following four stages [36]:

1. **Embedding**: A sliding window with length *L* forms a trajectory matrix, **X**, transforming the original one-dimensional series into a multidimensional series of lagged vectors.
2. **Decomposition**: Singular value decomposition (SVD) is applied to **X** to identify the principal components.
3. **Grouping**: Singular values are grouped into deterministic components (trend and periodic) and stochastic components (residuals).
4. **Reconstruction**: The final step involves reconstructing the time series through diagonally averaging grouped matrices.

Our study combines SSA with ML and DL techniques to capture both deterministic and stochastic aspects of HR dynamics, extending the SSA’s applications to HR prediction.

### 2.3. Recurrent Neural Networks (RNN)

RNNs are designed to handle sequential data, making them well-suited for HR forecasting. The architecture possesses feedback loops that retain information from previous time steps, capturing temporal patterns in time series data.

A typical RNN has hidden states $h_{t}$ that update at each time step *t* as follows [37]:

$$h_{t}=\sigma \left(W_{xh}x_{t}+W_{hh}h_{t-1}+b_{h}\right)$$

where $W_{xh}$ and $W_{hh}$ are weight matrices for the input and hidden state; $b_{h}$ is a bias term; and σ is an activation function, often a tanh or sigmoid function.

The output ${\hat{y}}_{t}$ is computed as follows:

$${\hat{y}}_{t}=W_{hy}h_{t}+b_{y}$$

where $W_{hy}$ is the weight matrix for the hidden-to-output layer, and $b_{y}$ is the output bias.

RNN are trained using backpropagation through time (BPTT), though they face challenges with long-term dependencies, which variants like LSTM address [37].

### 2.4. Long Short-Term Memory (LSTM)

LSTM, a specialized RNN, effectively manages long-term dependencies by incorporating memory cells and gating mechanisms [38,39]. The cell includes the following:

- **Forget Gate** $f_{t}$: Decides what portion of the cell state $C_{t-1}$ should be forgotten.
  $$f_{t}=\sigma \left(W_{xf}x_{t}+W_{hf}h_{t-1}+b_{f}\right)$$
- **Input Gate** $i_{t}$: Determines new information to add to the cell state.
  $$i_{t}=\sigma \left(W_{xi}x_{t}+W_{hi}h_{t-1}+b_{i}\right)$$
  with a candidate cell state as follows:
  $${\tilde{C}}_{t}=\tanh\left(W_{xc}x_{t}+W_{hc}h_{t-1}+b_{c}\right)$$
- **Output Gate** $o_{t}$: Controls what part of the cell state flows to the hidden state $h_{t}$.
  $$o_{t}=\sigma \left(W_{xo}x_{t}+W_{ho}h_{t-1}+b_{o}\right)$$

The cell state $C_{t}$ and hidden state $h_{t}$ are updated as follows:

$$C_{t}=f_{t}\circ C_{t-1}+i_{t}\circ {\tilde{C}}_{t}$$

$$h_{t}=o_{t}\circ \tanh\left(C_{t}\right)$$

where ∘ denotes element-wise multiplication. These gates allow LSTMs to handle both short- and long-term dependencies, making them ideal for HR predictions.

### 2.5. One-Dimensional Convolutional Neural Networks (1D CNN)

1D CNNs are adapted for time series prediction due to their ability to capture complex temporal dependencies. Their architecture includes convolutional, pooling, and fully connected layers [40].

Each convolutional layer applies a kernel κ[n] to the input time series x[n], a s follows:

$$\zeta \left[n\right]=x\left[n\right]*\kappa \left[n\right]=\sum_{m=0}^{\vartheta -1}\kappa \left[m\right]\cdot x\left[n-m\right]$$

where ∗ denotes the convolution operation and ϑ is the kernel size.

The CNN output is passed through an activation function such as the following:

$$R\left(\zeta \right)=\left\{\begin{array}{cc} \zeta & \mathrm{if}\;\zeta >0\\ \alpha \cdot (\exp(\zeta )-1) & \mathrm{if}\;\zeta \leq 0 \end{array}\right.$$

Pooling layers reduce dimensionality, and fully connected layers produce the final output. This architecture captures temporal patterns, making it suitable for HR prediction.

### 2.6. Physics-Informed Neural Networks (PINNs)

PINNs incorporate physical laws into the ML framework, enhancing prediction accuracy by reducing dependence on large datasets [41]. Physiological dynamics are embedded using Taylor approximations for HR prediction, calculating residuals between model predictions and expected HR behavior.

The total loss $L_{t}$ is a weighted sum of conventional supervised loss $L_{c}$ and physics-based loss $L_{p}$:

$$L_{t}=\alpha L_{c}+\beta L_{p},$$

where

- $L_{c}$ measures the error between predictions and ground truth HR labels.
- $L_{p}$ enforces adherence to the physiological dynamics.
- α and β are weighting factors balancing the accuracy with the physical constraints.

#### Hybrid SSA-ML/DL Models

**Figure 2:**
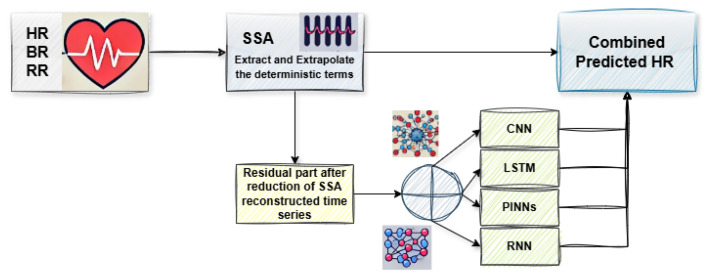
Schematic of Prediction algorithm.

We implemented a hybrid approach combining SSA with various ML/DL models. As can be seen in Figure 2, the SSA component decomposes the time series into deterministic and stochastic components, enabling the ML/DL models to focus on the residual patterns. The general framework of our approach is as follows:

1. **SSA Decomposition and Prediction**: Apply SSA to the input time series to decompose it into deterministic components, including trend and periodic patterns, as well as residuals. Then, the deterministic part is extrapolated using SSA.
2. **Residual Prediction with ML/DL**: Use an ML/DL model to predict the residual component, capturing complex, nonlinear relationships in the time series data.
3. **Combined predicted HR**: Combine the SSA-derived deterministic components with the ML/DL-predicted residuals to produce the final HR prediction, leveraging predictable trends and learned residual patterns.

This hybrid strategy leverages SSA’s strength in extracting deterministic trends and periodic patterns while allowing ML/DL models to focus on the residual components that require more sophisticated learning capabilities.

We implemented the following hybrid models in this study:

- **SSA + LSTM**.
- **SSA + 1D CNN**.
- **SSA + PINNs**.
- **SSA + RNN**.

### 2.7. Model Training and Evaluation

The models were implemented in **MATLAB (R2024a)** using the Machine Learning and Deep Learning Toolboxes. Each hybrid model was trained using different input parameter combinations as follows: HR only, HR + BR, HR + RR, and HR + BR + RR. The dataset was split 80:20 for training and testing. Mean absolute error (MAE) was used to evaluate the performance, expressed as follows:

(1) $$MAE=\frac{1}{n}\sum_{i=1}^{n}\left|y_{i}-{\hat{y}}_{i}\right|$$

where $y_{i}$ are the true values and ${\hat{y}}_{i}$ are the predicted values. Lower MAE values indicate higher prediction accuracy.

## 3. Results

Our investigation into HR prediction using various ML techniques and input parameter combinations yielded several significant findings. We used MAE as the primary metric to assess prediction accuracy.

### 3.1. Model Performance

**Figure 3:**
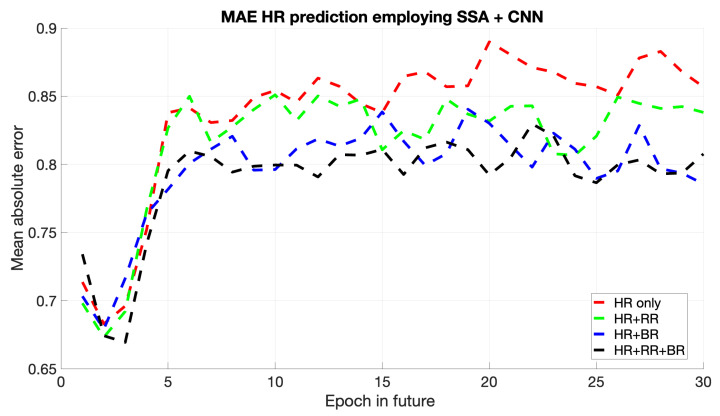
Mean of HR’s MAE prediction for the next epochs using SSA + CNN.

The MAE comparison for the four hybrid models, SSA + CNN, SSA + LSTM, SSA + PINNs, and SSA + RNN, reveals distinct performance characteristics across methods. As shown in Figure 3, the CNN model effectively captures local temporal patterns, maintaining consistent performance overall, though minor fluctuations in MAE appear across specific intervals, while CNNs are proficient at learning spatially correlated features, they face challenges in modeling long-term dependencies, as reflected by periodic increases in MAE.

**Figure 4:**
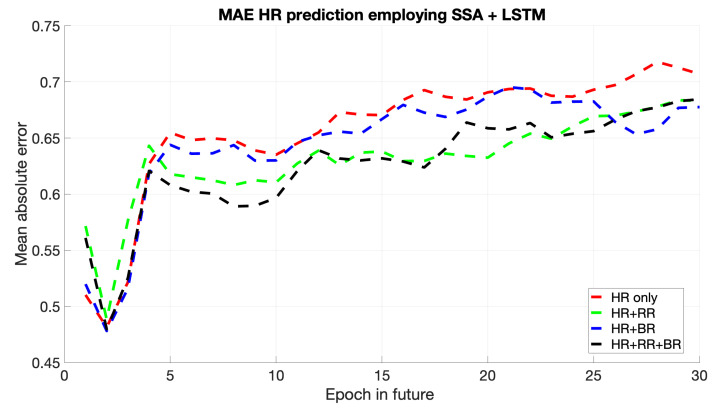
Mean of HR’s MAE prediction for the next epochs using SSA + LSTM.

The error plot in Figure 4 indicates that the LSTM model achieves lower error rates than other combinations. The LSTM’s capacity for retaining long-term dependencies improves accuracy and produces a more stable MAE curve. This suggests that the SSA + LSTM model is appropriate for time series data with complex temporal dynamics.

**Figure 5:**
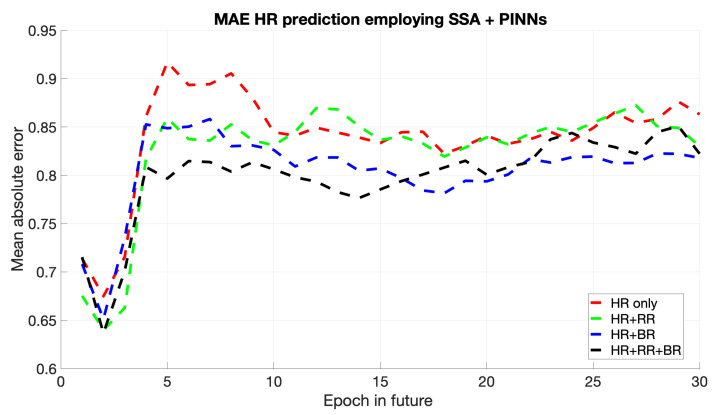
Mean of HR’s MAE prediction for the next epochs using SSA + PINNs.

The MAE plot for SSA + PINNs, shown in Figure 5, demonstrates competitive performance, especially in intervals where physical constraints closely align with observed data patterns. Although the overall MAE is comparable to that of the LSTM model, the PINNs approach benefits from lower error margins in regions where the physical model dominates. Minor fluctuations in MAE occur in data-driven regions with less applicable physical laws. However, PINNs effectively enhance model interpretability and accuracy when physical information is available.

**Figure 6:**
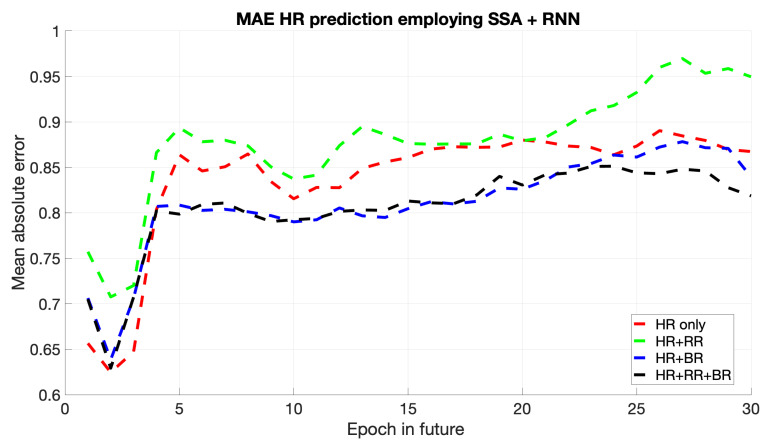
Mean of HR’s MAE prediction for the next epochs using SSA + RNN.

In Figure 6, the RNN model shows relatively low error rates, performing well in the short term and effectively capturing immediate temporal dependencies. If the prediction horizon increases, the MAE does, too; thus, the RNN may be incapable of holding long-term dependencies compared to the LSTM. That would imply that SSA + RNN, while fitting for short-term forecasting, might not be able to grasp a complex structure in a more extended sequence.

SSA + LSTM has consistently yielded the minimum MAE among the different models, capturing both the dataset’s short- and long-term dependencies. Although the SSA + CNN has demonstrated excellent performance in recognizing local temporal features, its inability to perform as well for some intervals evidences that it is limited to model long-term dependencies. It gives a competitive performance for the SSA + PINNs approach for the region dominated by physical dynamics and, hence, would be suitable in domains reliant on domain knowledge. Finally, although the SSA + RNN model was effective regarding short-term predictions, its tendency to grow with longer horizons depicts an inherent shortfall in handling long-term temporal patterns.

### 3.2. Impact of Input Parameters

We assessed the impact of various ECG parameter combinations on prediction accuracy by comparing the configurations HR + BR, HR + RR, and HR + BR + RR against HR alone (see Table 2). Our analysis revealed the following:

- The HR + BR + RR combination consistently outperformed all other input configurations across the tested models, aligning with previous findings that highlight the value of incorporating multiple physiological inputs.
- While HR + BR and HR + RR both improved prediction accuracy compared to HR alone, they were less effective than the comprehensive HR + BR + RR combination.
- Including BR and RR data added valuable information, resulting in more accurate predictions.

**Table 2:** Parameter impact analysis.

Model	HR + BR	HR + RR	HR + BR + RR
SSA + CNN	5.09	2.54	5.90
SSA + LSTM	2.04	4.38	4.99
SSA + PINNs	4.23	1.73	4.99
SSA + RNN	3.39	-4.66	4.03

### 3.3. Comparison of Standalone ML and SSA-Enhanced Models

Our analysis reveals that when standalone ML models exhibit higher prediction errors, particularly in the early stages of future epoch predictions, the hybrid SSA-enhanced models demonstrate significantly improved accuracy and stability.

**Figure 7:**
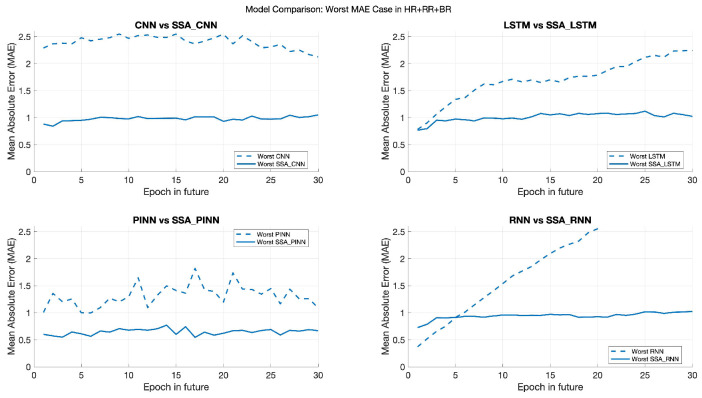
Mean absolute error (MAE) comparison of standalone machine learning (ML) models and SSA-enhanced models in the worst case scenario.

**Figure 8:**
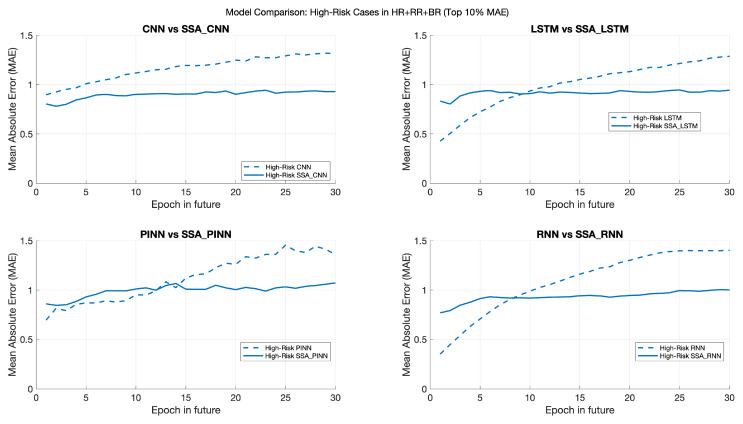
Comparison of prediction errors for standalone ML models and SSA-enhanced models in high-risk samples (90th percentile MAE).

As depicted in Figure 7 and Figure 8, the standalone ML models (represented by dashed lines) show a tendency for error to increase as predictions move further into the future. In contrast, the hybrid models (solid lines) maintain lower and more consistent error rates, particularly for samples with a higher probability of error.

Specifically, we have focused on samples falling within the 90th percentile of prediction error for the standalone models. Figure 7 and Figure 8 show that the hybrid models consistently outperform the standalone models in this high-risk category. For instance, the MAE for the hybrid models remains below 1 (and slightly above 1 for SSA-PINNs), while the standalone models exhibit MAE values ranging from 1.3 to 1.5.

The proposed hybrid model effectively mitigates sample prediction errors with a higher likelihood of inaccuracies, highlighting this approach’s added value. Since wearable sensors are intended for continuous monitoring and early warning, hybrid models’ improved stability and accuracy in high-risk scenarios make them more reliable for critical applications. For individuals with stable health and lower prediction error probabilities, standalone ML models may offer sufficient performance. However, our study primarily focuses on the impact of incorporating SSA for improved accuracy in high-risk predictions and the value of adding additional measurements (HR + RR + BR) to the model.

### 3.4. Computational Time Analysis

While a comprehensive efficiency analysis was within the scope of this study, we can provide some insights into the computational overhead introduced by the SSA component. As expected, the incorporation of SSA into the machine learning pipelines generally resulted in an increase in training time. For example, the total time cost for training, testing, and prediction across our 81 participant samples increased from 12,545 s for the RNN model to approximately 14,653 s for the SSA-RNN variant. This represents an average increase of approximately 2107 s across all 81 samples or roughly 26 s per sample. In fact, each gadget would predict the heart rate individually, so the level of complexity will be far lower than the current study. It is crucial to note that this time cost encompasses the entire training, testing, and prediction process. Furthermore, most of this time is attributed to the training phase, while the prediction phase is significantly faster for only a moment. For instance, dividing 14,653 s by 81 samples yields approximately 181 s per sample for the entire process. However, this includes the training time, typically a one-time operation performed periodically for model updates. The model would be pre-trained and deployed in a real-world wearable device scenario, with only the prediction phase being executed on the device itself. Therefore, the actual computational burden during real-time operation would be substantially lower.

## 4. Discussion

The results of this study provide valuable insights into applying ML techniques for HR prediction, especially in real-time operating data from wearable sensors. Our findings confirm the effectiveness of the hybrid SSA-LSTM model, which combines SSA with LSTM networks, in capturing complex temporal dependencies and patterns in HR data. We also determined that incorporating diverse cardiovascular variables like heart rate, breathing rate, and RR interval data may significantly improve prediction accuracy. The superior performance of the SSA-LSTM model for various input configurations generally underlines its robustness and practicality for physiological time series applications. In this regard, the preprocessing step by SSA is highly effective due to the following reasons:

- SSA decomposes the time series into distinct components—trend, periodicity, and noise—enabling the LSTM to focus on learning critical patterns. By isolating noise and periodic elements, SSA enhances signal quality, thereby improving the LSTM’s ability to model underlying HR dynamics [33,34].
- SSA’s noise reduction likely contributes to enhanced model generalization. Physiological data from wearables can be noisy due to sensor inaccuracies or user movement; SSA mitigates this noise, resulting in improved predictive performance and more reliable HR monitoring across diverse conditions [12].
- Known for its strength in modelling long-term dependencies in time series data, the LSTM benefits from SSA’s preprocessing by concentrating on long-term trends after irrelevant or noisy components are removed, making it especially effective for HR prediction [34].

Using the SSA with the LSTM model helps us better understand heart rate changes. This method improves how we analyse data from wearable sensors and shows us the potential of this advanced model in our research. Our study demonstrates that integrating heart rate, blood rate, and respiration rate data enhances the model’s performance due to their connection to the body. For instance, respiratory sinus arrhythmia is a variation of HR according to the breathing pattern; hence, it induces cyclic variations in HR synchronized with the respiratory cycle. Similarly, RR interval data reflect variability in heart rate and thus carry information about the health status of the cardiovascular system and the activity of the autonomic nervous system [15]. Incorporating multiple variables provides a deeper understanding of the physiological processes influencing heart rate. The multivariate approach is necessary because a univariate focus on heart rate might miss important interrelationships and even lower the prediction accuracy. This improved performance by combining HR, BR, and RR strengthens the justification for using multivariate data for accurate physiological signal predictions. The significant reduction in the MAE of the SSA-LSTM model highlights its clinical value. Accurate HR estimates are critical in monitoring cardiac health. The early detection of abnormalities, supported by its performance assessment [28], can identify potential patient conditions and allow for timely treatment. For athletes, It can increase the usefulness of wearable devices. It provides real-time insights into activity, recovery, and health [29]. Multivariate HR prediction promotes personalized healthcare and early warning systems for cardiovascular events. The proposed HR prediction models could be important for health and sports applications. In sports performance, accurate HR prediction can assist in monitoring recovery states, detecting training load thresholds, and preventing overtraining syndromes. In healthcare, these models can assist in the early detection of cardiovascular anomalies, enabling timely interventions for individuals with heart conditions. While promising, the following limitations must be addressed to enhance the generalizability and real-world applicability of our findings:

- This research relied on a specific dataset, which may limit applicability to other populations or settings. Further research should validate these findings across diverse demographics, age ranges, and conditions. It is also recommended that various physical activities, stress levels, climate, variables, and environments be included. Large-scale validation studies with heterogeneous data can help ensure broader model generalizability [30].
- While our study primarily focused on predictive accuracy, computational efficiency remains a limitation. The SSA-LSTM model, in particular, is computationally intensive due to its sequential processing, which may hinder real-time deployment in low-resource environments. Future work should systematically analyse model efficiency and explore optimization strategies such as model compression, pruning, and hardware acceleration [32].
- Our study mainly focuses on short-term HR forecasting. Evaluating model performance for longer time horizons, such as hourly or daily forecasts. It may be beneficial for chronic disease management and long-term fitness monitoring.
- Although we focused on HR, BR, and RR intervals, additional parameters, such as skin temperature, galvanic skin response, and blood oxygen levels, may offer further insights into cardiovascular health. Examining the impact of these variables on HR prediction accuracy could lead to more comprehensive models that assess a wider range of health metrics [3].
- The complexity of the SSA-LSTM model may limit its interpretation and its ability to achieve high accuracy. It is therefore challenging to understand the contribution of individual physiological parameters or components to the final prediction. Future research should develop ways to increase model transparency [34].
- While HR monitoring is valuable, it may not fully capture aerobic capacity in high-intensity exercise, where $VO_{2}$ max plays a key role. Since our dataset does not include $VO_{2}$ max, this study does not explore its relationship with HR. Future research should incorporate additional physiological markers, such as lactate threshold, to improve HR prediction models for high-intensity training.
- The primary computational cost associated with the offline training phase. While real-time prediction on the wearable device is significantly faster, ensuring practical applicability, and while SSA integration increases training time, this is a one-time process; prediction, the crucial on-device operation, remains computationally efficient. Despite the training overhead, the prediction phase, essential for wearable device functionality, exhibits rapid execution, mitigating concerns about real-time performance. Investigating the computation cost of real-time monitoring and prediction in future studies is beneficial.

These findings build upon and extend existing research. For instance, previous studies have demonstrated the utility of LSTM for HR prediction but have yet to explore the potential of integrating LSTM with SSA or merging multivariate physiological inputs [19,35]. Correspondingly, studies have scrutinized the role of BR in HR prediction but have not yet considered the added value of RR interval data or the hybrid SSA-LSTM approach. The uniform performance improvement when fusing HR, BR, and RR interval data underscores the essence of multivariate modelling for physiological signal prediction.

## 5. Conclusions

This study is devoted to improving heart rate prediction accuracy by integrating Singular Spectrum Analysis (SSA) into machine learning methods. Our results provide insights for new healthcare and sports approaches, such as the early detection of cardiac abnormalities, tailored monitoring, and optimization of athletic performance. This research’s outcomes illustrate that the hybrid SSA-LSTM technique outperformed its rival, yielding the lowest error (in this case, the Mean Absolute Error—MAE) with greater efficiency. Furthermore, it has been proven that prediction performance significantly improves when more physiological parameters are considered in the prediction model, especially BR and RR. Although our results show promising developments, demonstrating that our proposed method and input variables can improve HR prediction, several challenges remain. These include computational complexity, the need for further validation, and the requirement for real-world implementation. Therefore, optimizing model efficiency, extending the prediction horizon, and assessing additional physiological metrics could enhance its applicability. Additionally, while our study focused on HR prediction using physiological signals, the dataset does not explicitly label training or extreme physical stress cases. However, physiological markers such as prolonged elevated HR and increased RR interval variability may indirectly indicate excessive strain. Future research should incorporate labelled datasets with stress indicators to improve risk assessment for athletes and individuals undergoing intensive training. Progress in these areas can contribute to the evolution of wearable health devices, supporting real-time, personalized healthcare solutions for cardiovascular monitoring and the early detection of potential health risks.

## Figures and Tables

**Table 1:** Descriptive characteristics of the study population.

Characteristic	Mean ± SD	Range
Age (years)	30 ± 13	18–45
Gender (M/F)	53/28	-
Height (cm)	170 ± 30	159–190
Weight (kg)	71 ± 21	50–90
Smoking (No/Yes)	39/29	-
Alcohol Consumption (No/Sometimes)	11/55	-
Weekly Training (hours)	4 ± 1	2–15
Sports Experience (%)	Beginner (30%), Intermediate (50%), Advanced (20%)	-

## Data Availability

The raw data used in this study are available at: Agnese Sbrollini, Micaela Morettini, Elvira Maranesi, Ilaria Marcantoni, Amnah Nasim, Roberta Bevilacqua, Giovanni R. Riccardi, and Laura Burattini. 2019. *Sport Database: Cardiorespiratory data acquired through wearable sensors while practicing sports.Data in Brief, 27:104793. Available online: https://doi.org/10.1016/j.dib.2019.104793.

## Abbreviations

The following abbreviations are used in this manuscript:

AR	Autoregressive
ARIMA	AutoRegressive Integrated Moving Average
ARMA	Autoregressive-Moving Average
BiLSTM	Bidirectional Long Short-Term Memory
BPTT	BackPropagation Through Time
BR	Breathing Rate
CNN	Convolutional Neural Network
DL	Deep Learning
ECG	Electrocardiogram
HR	Heart Rate
LSTM	Long Short-Term Memory
MAE	Mean Absolute Error
ML	Machine Learning
PINN	Physics-Informed Neural Network
RF	Random Forest
RNN	Recurrent Neural Network
RR	RR-Interval (time between consecutive heartbeats)
SVD	Singular Value Decomposition
SVM	Support Vector Machine
SSA	Singular Spectrum Analysis
